# The Prevalence and Malignancy Risk of Breast Incidental Uptake Detected by PET/CT with Different Radiopharmaceuticals: An Updated Systematic Review and Meta-Analysis

**DOI:** 10.3390/ph18121831

**Published:** 2025-12-01

**Authors:** Cesare Michele Iacovitti, Andreea Marin, Slavko Tasevski, Chiara Martinello, Marco Cuzzocrea, Gaetano Paone, Alessio Rizzo, Domenico Albano, Giorgio Treglia

**Affiliations:** 1 Division of Nuclear Medicine, Imaging Institute of Southern Switzerland, Ente Ospedaliero Cantonale, 6500 Bellinzona, Switzerland; cesaremichele.iacovitti@eoc.ch (C.M.I.); chiara.martinello@eoc.ch (C.M.); marco.cuzzocrea@eoc.ch (M.C.); gaetano.paone@eoc.ch (G.P.); 2 Clinic of Nuclear Medicine, Central University Emergency Military Hospital “Dr. Carol Davila”, 10825 Bucharest, Romania; andreea.marin0125@rez.umfcd.ro; 3 University Institute for Positron Emission Tomography, 1000 Skopje, North Macedonia; slavko.tasevski@uipet.mk; 4 Faculty of Biomedical Sciences, Università della Svizzera Italiana, 6900 Lugano, Switzerland; 5 Department of Nuclear Medicine, Candiolo Cancer Institute, FPO-IRCCS, 10060 Turin, Italy; alessio.rizzo@ircc.it; 6 Nuclear Medicine Department, ASST Spedali Civili, University of Brescia, 25123 Brescia, Italy; domenico.albano@unibs.it; 7 Faculty of Biology and Medicine, University of Lausanne, 1015 Lausanne, Switzerland

**Keywords:** breast, incidentaloma, incidental, PET, positron emission tomography, nuclear medicine, FDG, somatostatin, fluorocholine, hybrid imaging

## Abstract

**Background:** Meta-analyses on the prevalence and clinical significance of breast incidental uptake (BIU) at PET/CT are available only for [^18^F]FDG, showing that BIU is rare but malignant in a substantial proportion of cases. This study aimed to update the pooled prevalence and malignancy risk of BIU using different PET radiotracers, expanding [^18^F]FDG-based evidence. **Methods:** A comprehensive literature search of studies on BIU was carried out in two bibliographic databases, and the literature was screened up to 25 May 2025. Only original articles reporting BIU were selected. A proportion meta-analysis was conducted on a patient-based analysis using a random-effects model to estimate pooled prevalence, malignancy rate, and histological distribution. **Results:** In total, 29 studies were included in the systematic review and meta-analysis. PET/CT was performed using [^18^F]FDG (*n* = 25), radiolabeled somatostatin analogues (SSAs) (*n* = 3), or [^18^F]fluorocholine (*n* = 1). The pooled prevalence of BIU was 0.5% for [^18^F]FDG PET/CT, 3.4% for SSA PET/CT, and 2.6% for [^18^F]fluorocholine. The pooled malignancy rate among BIUs (female patients) was 33.5% for [^18^F]FDG, 86.4% for SSA, and 70% for [^18^F]fluorocholine PET/CT. Histological data were mainly available for [^18^F]FDG PET/CT, showing ductal carcinoma as the most frequent malignant histotype (pooled value 42.2%) and fibroadenoma (pooled value 14.8%) as the most frequent benign histotype. **Conclusions:** Similar to the case for [^18^F]FDG, BIU using other PET radiopharmaceuticals is uncommon but often malignant. Therefore, BIU should prompt dedicated breast imaging and, when indicated, histopathological confirmation. Further well-designed studies are needed to clarify the clinical impact of BIU detection and the prevalence and clinical significance of BIU using tracers other than [^18^F]FDG.

## 1. Introduction

Incidental imaging findings, often called incidentalomas, are lesions detected on examinations performed for indications unrelated to the organ imaged. Their observed frequency has risen in parallel with the wider use of advanced imaging and with improvements in spatial and contrast resolution [1,2]. Although many incidental findings prove clinically relevant, their prevalence and subsequent implications depend on both anatomical site and imaging technique [1,2].

Within molecular imaging, positron emission tomography (PET) has become integral to oncologic and non-oncologic care. PET is commonly combined with computed tomography (CT) or magnetic resonance imaging (MRI) as hybrid techniques, and several radiopharmaceuticals are available to evaluate distinct metabolic pathways or receptor profiles [3]. The most widely used tracer is [^18^F]fluorodeoxyglucose ([^18^F]FDG), a glucose analogue; however, other tracers, such as radiolabeled somatostatin analogues (SSAs) and [^18^F]fluorocholine, are increasingly used for specific clinical purposes [4]. In particular, the use of SSA and [^18^F]fluorocholine PET/CT has expanded beyond traditional indications, such as neuroendocrine tumors and prostate cancer, to include additional areas like parathyroid disorders for [^18^F]fluorocholine and meningiomas for SSAs, thus broadening the patient populations undergoing these scans [2,3].

Incidental focal uptake in the breast on PET/CT (breast incidental uptake, BIU) is a well-known occurrence in everyday practice. Despite organized breast-cancer screening with mammography, unsuspected lesions may be encountered on PET/CT studies acquired for non-breast indications [5,6]. Because [^18^F]FDG uptake is not tumor specific and may also occur in benign inflammatory or hyperplastic conditions, the mere presence of focal activity does not equate to malignancy. Conversely, a considerable proportion of BIUs represent previously unrecognized breast cancer, making accurate estimates of prevalence and malignancy risk essential to guide subsequent diagnostic work-up and to avoid both over- and under-management [5,6].

Previous reviews have mainly focused on BIU detected by [^18^F]FDG-PET/CT in women, reporting variable prevalence and malignancy rates across studies [5,6]. However, the clinical landscape has evolved: PET technology and usage have expanded, and incidental uptakes are now reported also with tracers beyond [^18^F]FDG, including SSAs (in the setting of neuroendocrine tumors) and [^18^F]fluorocholine (in parathyroid adenoma localization, prostate-cancer imaging, and other indications) [2,4,7]. The prevalence and clinical significance of BIU may differ by tracer because of distinct biological targets and background biodistribution [4].

We conducted an updated systematic review and meta-analysis to estimate the pooled prevalence of BIU on PET/CT across tracers, overall and in women; to quantify the pooled malignancy rate among BIUs (overall and among histologically verified lesions); and to describe the histopathologic spectrum of malignant and benign BIUs. We hypothesized that both prevalence and risk of malignancy vary according to tracer type.

## 2. Materials and Methods

### 2.1. Review Protocol, Working Group, and Review Question

This review followed a predefined protocol [8] and is reported in accordance with PRISMA 2020 [9]. Registration on PROSPERO was not performed, consistent with PRISMA guidance that registration is recommended but not mandatory [9].

Three reviewers (C.M.I., A.M., and G.T.) independently performed screening and selection; disagreements were resolved by discussion with senior authors via videoconference. The review question was defined a priori as follows: “What are the prevalence and clinical significance of breast incidental uptake (BIU) at PET/CT across radiopharmaceuticals?”.

### 2.2. Search Strategy

A comprehensive search was conducted in two bibliographic databases (PubMed/MEDLINE and Cochrane Library) until May 2025. The search string combined free-text terms reflecting the organ, the incidental nature of findings, and the imaging modality:

(A) “breast” OR “mammary” AND (B) “incidental” OR “incidentaloma*” OR “incidental*” OR “unexpected” OR “unusual” AND (C) “PET” OR “positron”.

No language or date limits were applied. To enhance sensitivity, the reference lists of eligible studies and relevant reviews were hand-searched for additional records.

### 2.3. Study Selection

Eligibility criteria were prespecified. The inclusion criteria were as follows: original articles reporting prevalence and/or clinical significance of BIU detected on PET/CT using different radiopharmaceuticals performed for indications unrelated to primary breast disease. The exclusion criteria were as follows: articles outside the scope, articles lacking extractable data on BIU prevalence or malignancy, reviews, editorials, comments, letters, and case reports.

Titles/abstracts obtained with the predefined strategy were screened independently by three reviewers (C.M.I.; A.M.; G.T.). Full texts of potentially relevant records were then assessed. Final inclusion was established by consensus. Studies were entered into the quantitative analysis (meta-analysis) only when sufficient data were available to calculate at least one prespecified proportion (e.g., number of BIUs over PET/CT examinations, or number of malignant BIUs over histologically verified BIUs).

### 2.4. Data Extraction and Quality Assessment

Three reviewers (C.M.I.; A.M.; G.T.) independently extracted data using standardized forms: basic study characteristics; PET tracer; study population and sex distribution; number of PET/CT examinations; number of BIUs overall and in women; number of BIUs that underwent histopathology (BIU-H); number of malignant BIUs; and histopathologic diagnoses (malignant and benign subtypes). Discrepancies were resolved by consensus. Risk of bias and study quality were evaluated with the NIH Quality Assessment Tools online appropriate to each design.

### 2.5. Statistical Analysis

We performed proportion meta-analyses using random-effects models to account for between-study variability. Results are reported as pooled proportions with 95% confidence intervals. Heterogeneity was quantified with I^2^. Prespecified subgroup analyses included different radiopharmaceuticals. Sensitivity analyses were planned to exclude studies with evident selection bias from pooling, while retaining them in the qualitative synthesis. Publication bias/small-study effects were explored visually with funnel plots and formally with Egger’s regression test (α = 0.05). Analyses were executed with the web platform MetaAnalysisOnline (https://metaanalysisonline.com/, last accessed on 14 November 2025, module “Proportion”; forest and funnel plots generated with default settings).

## 3. Results

### 3.1. Literature Search

**Figure 1:**
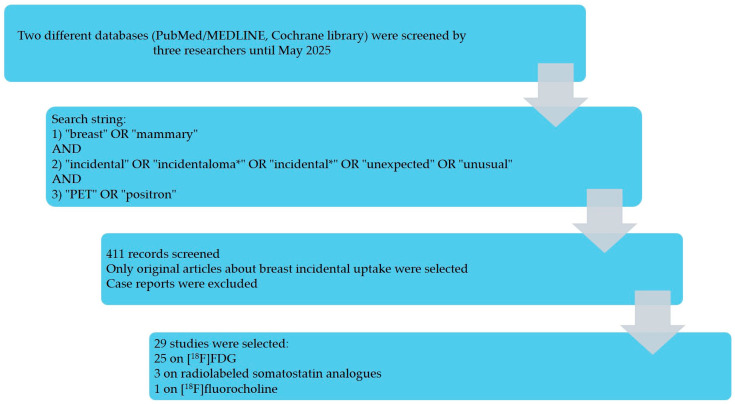
Results of the literature search.

The selection process is summarized in Figure 1. The search of two databases (PubMed/MEDLINE and the Cochrane Library) yielded 411 records screened by three reviewers. After title/abstract screening and full-text assessment, 29 studies met the inclusion criteria [10,11,12,13,14,15,16,17,18,19,20,21,22,23,24,25,26,27,28,29,30,31,32,33,34,35,36,37,38]: 25 used [^18^F]FDG [10,11,12,13,14,15,16,17,18,19,20,21,22,23,24,25,26,27,28,29,30,31,32,33,34], 3 used somatostatin receptor tracers (SSAs) [35,36,37], and 1 used [^18^F]fluorocholine [38]. Case reports and non-original articles were excluded. One [^18^F]FDG study with selection bias (Benveniste et al. [23]) was retained for the narrative synthesis but excluded from meta-analysis. Most of the included studies were retrospective.

### 3.2. Qualitative Synthesis

Table 1 summarizes the main characteristics and outcomes of the 29 included studies on breast incidental uptake (BIU) detected with PET tracers. In this review, BIU was defined as incidentally detected focal breast uptake on PET/CT performed for non-breast indications. Across studies, clinical indications, sex distribution, and verification pathways (biopsy vs. imaging follow-up) differed. When tissue diagnosis was obtained, a substantial proportion of BIUs proved malignant, with higher rates reported in SSA and fluorocholine series, and the most frequent malignancy was ductal carcinoma, whereas benign outcomes most often included fibroadenoma and other non-specific benign lesions.

#### 3.2.1. [^18^F]FDG PET/CT

Across studies, BIU on all scans was uncommon (pooled prevalence 0.5%) with higher values observed in female-only cohorts (pooled prevalence 1%). The outlier series by Benveniste et al. [23] (22.5% BIU) reflected referral/selection bias and was not included in meta-analysis. Histology was available for a large subset of [^18^F]FDG-BIUs, allowing pooled estimates of the malignant and benign spectrum. Among malignant BIUs, ductal carcinoma (DC) was the most frequent diagnosis, followed by other/unspecified primary malignancies, metastases, ductal carcinoma in situ (DCIS), and lobular carcinoma (LC); among benign entities, other/unspecified benign lesions and fibroadenoma predominated (Table 2).

#### 3.2.2. Somatostatin Receptor PET/CT (SSA)

Three eligible studies using DOTA-peptides were identified. SSA exams were mostly performed for neuroendocrine tumor work-up. Reported BIU prevalence ranged from 0.8% to 12.1% of scans overall, with higher values in smaller female-only cohorts (pooled prevalence 5.2%). Histology was available in a minority of BIUs; when obtained, malignancy among biopsied BIUs was frequent; however, the corresponding estimates showed wide confidence intervals due to the small number of contributing studies and events.

#### 3.2.3. [^18^F]Fluorocholine PET/CT

One study (Broos et al. [38]) provided extractable data. BIU prevalence was 2.6% overall and 3.4% among women; all 7/7 BIUs with histopathologic confirmation were malignant. Because only a single study was available, no pooling was performed for fluorocholine.

#### 3.2.4. Overall Study Quality

Using the NIH tools, the overall quality was moderate, mainly limited by retrospective design, variable verification with histology, and incomplete sex-specific denominators in some cohorts.

### 3.3. Quantitative Synthesis

#### 3.3.1. Prevalence of BIU

**Figure 2:**
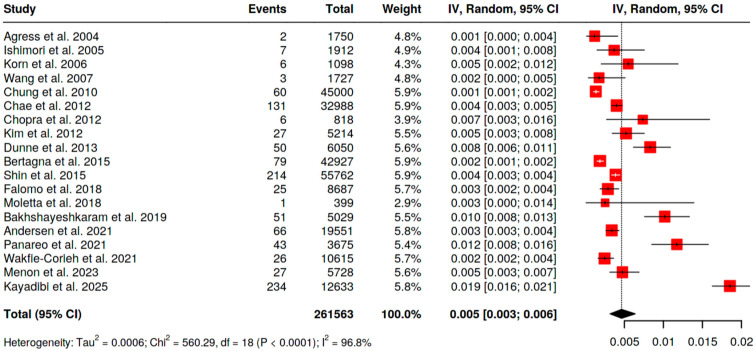
Pooled prevalence of BIU in both genders [10,11,12,13,16,18,19,20,21,24,26,27,28,29,30,31,32,33,34].

**Figure 3:**
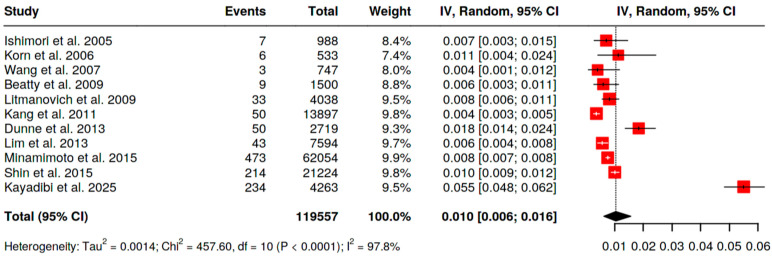
Pooled prevalence of BIU in female patients [11,12,13,14,15,17,21,22,25,26,34].

With [^18^F]FDG PET/CT the pooled prevalence of BIU across 19 analyzable series (*n* = 261,563 scans; Figure 2) was 0.5% (95% CI 0.3–0.6). Statistical heterogeneity was high (I^2^ = 96.8%), reflecting differences in populations and referral indications. Restricting to women (*n* = 119,557 scans; Figure 3), pooled BIU prevalence was 1.0% (95% CI 0.6–1.6), again with substantial heterogeneity (I^2^ = 97.8%).

With SSA PET/CT, based on three small studies, pooled prevalence was 3.4% (95% CI 0.0–11.5) with considerable heterogeneity (I^2^ = 72.4%). Female-only prevalence was 5.2% (95% CI 0.1–14.7; I^2^ = 55.9%). The wide CIs reflect sparse data and between-study variation.

With [^18^F]fluorocholine PET/CT the single eligible study reported 2.6% overall and 3.4% BIU in women (no heterogeneity measure applicable).

**Table 2:** Histological findings of breast incidentalomas at PET/CT.

PET Tracer	First Author [Ref.]	BIU-H	DC	% of DC Among BIU-H	DCIS	% of DCIS Among BIU-H	LC	% of LC Among BIU-H	OUM	% of OUM Among BIU-H	MET	% of MET Among BIU-H	FA	% of FA Among BIU-H	OBL	% of OBL Among BIU-H
**[^18^F]FDG**	Agress et al. [10]	2	1	50	-		-		1	50	-		-		-	
	Ishimori et al. [11]	2	2	100	-		-		-		-		-		-	
	Korn et al. [12]	6	5	83.3	-		-		-		-		1	16.7	-	
	Wang et al. [13]	3	2	66.7	-		-		-		-		1	33.3	-	
	Beatty et al. [14]	8	5	62.5	-		-		-		-		2	25	1	12.5
	Litmanovich et al. [15]	21	7	33.3	-		2	9.5	-		3	14.3	2	9.5	7	33.3
	Chung et al. [16]	12	6	50	-		-		-		1	8.3	1	8.3	4	33.3
	Kang et al. [17]	35	14	40	2	5.7	-		-		2	5.7	1	2.9	16	45.7
	Chae et al. [18]	60	23	38.3	3	5	1	1.7	-		5	8.3	8	13.3	20	33.3
	Chopra et al. [19]	3	-		-		-		2	66.7	-		-		1	33.3
	Kim et al. [20]	23	10	43.5	3	13	-		-		2	8.7	2	8.7	6	26.1
	Dunne et al. [21]	23	8	34.8	2	8.7	1	4.3	4	17.4	2	8.7	4	17.4	2	8.7
	Lim et al. [22]	17	5	29.4	3	17.6	-		1	5.9	-		2	11.8	6	35.3
	Benveniste et al. [23]	55	19	34.5	1	1.8	1	1.8	4	7.3	12	21.8	-		18	32.7
	Bertagna et al. [24]	35	17	48.6	2	5.7	4	11.4	-		2	5.7	9	25.7	1	2.9
	Minamimoto et al. [25]	161	95	59	27	16.8	-		39	24.2	-		-		-	
	Shin et al. [26]	60	21	35	1	1.7	1	1.7	2	3.3	2	3.3	10	16.7	23	38.3
	Falomo et al. [27]	11	-		-		-		-		-		-		-	
	Moletta et al. [28]	1	1	100	-		-		-		-		-		-	
	Bakhshayeshkaram et al. [29]	22	6	27.3	-		3	13.6	-		1	4.5	4	18.2	8	36.4
	Andersen et al. [30]	40	26	65	-		1	2.5	3	7.5	4	10	3	7.5	2	5
	Panareo et al. [31]	22	9	40.9	-		-		3	13.6	4	18.2	6	27.3	1	4.5
	Wakfie-Corieh et al. [32]	23	-		-		1	4.3	8	34.8	-		-		14	60.9
	Menon et al. [33]	26	10	38.5	2	7.7	1	3.8	3	11.5	2	7.7	4	15.4	4	15.4
	Kayadibi et al. [34]	63	10	15.9	-		2	3.2	3	4.8	5	7.9	21	33.3	22	34.9
Pooled values (95%CI)				**42.2%** (34–50.5)		**7.1%** (3.4–11.8)		**3.5%** (1.7–5.8)		**11.1%** (4.8–19)		**8.7%** (6–11.6)		**14.8%** (10.3–19.9)		**25.3%** (17.3–34)
**Radiolabeled** **Somatostatin** **analogues**	Elgeti et al. [35]	4	2	50	-		-		-		2	50	-		-	
	Kuyumcu et al. [36]	-	-		-		-		-		-		-		-	
	Cavicchioli et al. [37]	1	-		-		-		-		1	100	-		-	
**[^18^F]F-choline**	Broos et al. [38]	7	3	42.9	1	14.3	2	28.6	1	14.3	-		-		-	

Legend: BIU-H = number of breast incidentalomas that underwent histology, DC = ductal carcinoma, DCIS = ductal carcinoma in situ, LC = lobular carcinoma, OUM = other or unspecified primary malignant lesions, MET = metastatic lesion, FA = fibroadenoma, OBL = other or unspecified benign lesion.

#### 3.3.2. Risk of Malignancy

**Figure 4:**
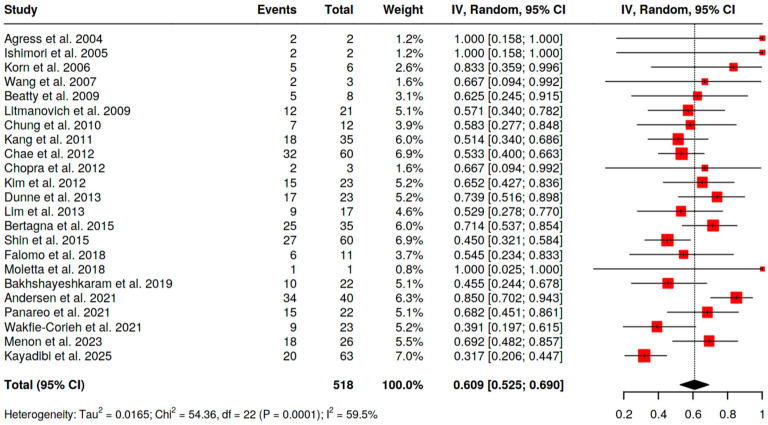
Pooled prevalence of MBIU among BIU-H [10,11,12,13,14,15,16,17,18,19,20,21,22,24,26,34].

Among histologically verified BIUs (BIU-H),

− For [^18^F]FDG PET/CT, the pooled malignancy risk was 60.9% (95% CI 52.5–69.0; I^2^ = 59.5%) (Figure 4);
− For SSA PET/CT, the malignancy risk was 100% (95% CI 66.8–100; I^2^ = 0%), acknowledging very small numbers;
− For [^18^F]fluorocholine PET/CT, the malignancy risk was 100% (7/7 malignant lesions in a single study).

**Figure 5:**
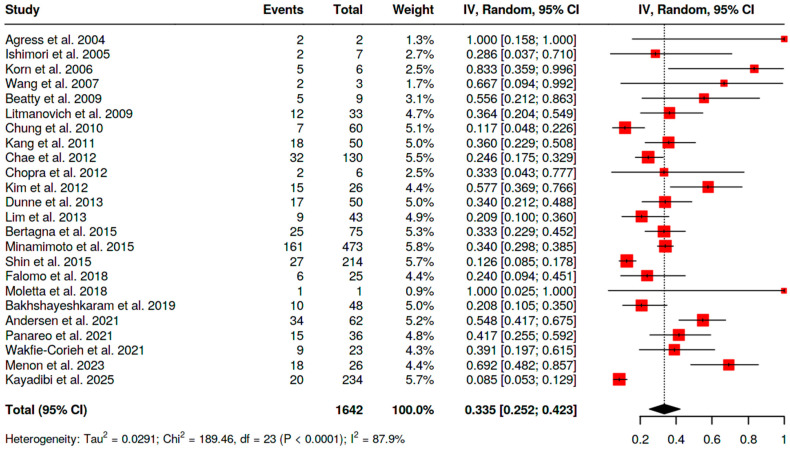
Overall risk of malignancy of BIU in female patients [10,11,12,13,14,15,16,17,18,19,20,21,22,24,25,26,27,28,29,30,31,32,33,34].

Overall malignancy risk among BIUs in women (i.e., malignant BIUs/total BIUs in female cohorts) was as follows:

− For [^18^F]FDG PET/CT, 33.5% (95% CI 25.2–42.3; I^2^ = 87.9%) (Figure 5);
− For SSA PET/CT, 86.4% (95% CI 72.0–100; I^2^ = 50.7%);
− For [^18^F]fluorocholine PET/CT, 70% (single study).

**Figure 6:**
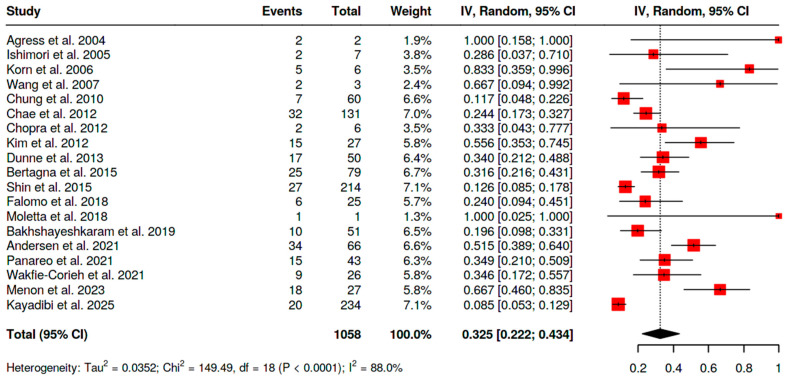
Overall risk of malignancy of BIU in both genders [10,11,12,13,16,18,19,20,21,24,26,27,28,29,30,31,32,33,34].

The overall risk of malignancy in BIU in both genders (Figure 6) was similar to that observed in the female cohort, but the analysis had reduced statistical power due to limited data. For [^18^F]FDG PET/CT this value was 32.5% (95% CI 22.2–43.4; I^2^ = 88%).

#### 3.3.3. Histopathology

Where pooling histology was possible (Table 2), in particular with [^18^F]FDG PET/CT for which more data were available, malignant BIUs were predominantly ductal carcinoma (42.2%), with DCIS (7.1%) and LC (3.5%) being less frequent; other/unspecified primary malignancies comprised 11.1%, and metastases were 8.7%. Among benign lesions, fibroadenoma accounted for 14.8% and other/unspecified benign lesions for 25.3%.

#### 3.3.4. Heterogeneity, Sensitivity Analyses, and Potential Biases

Across prevalence and malignancy outcomes, statistical heterogeneity was frequent, especially for [^18^F]FDG prevalence (I^2^ > 90%) and for the overall risk of malignancy among BIUs in women. The main contributors were differences in population mix (oncologic or non-oncologic indications; screening backgrounds), sex-specific denominators (often incomplete), verification bias (variable histology rates), and heterogeneity in data reporting (e.g., counts given per scan vs. per patient). Excluding the selection-biased [^18^F]FDG study (Benveniste et al. [23]) did not materially change the pooled estimates.

**Figure 7:**
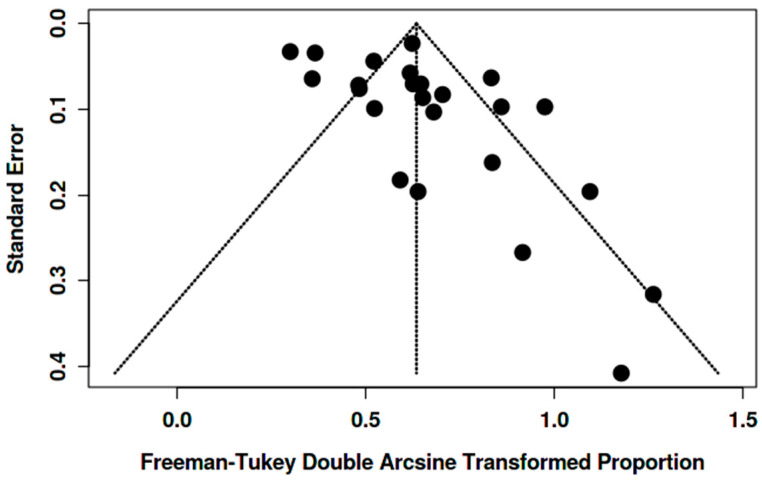
Funnel plot for overall risk of malignancy among BIUs (women).

**Figure 8:**
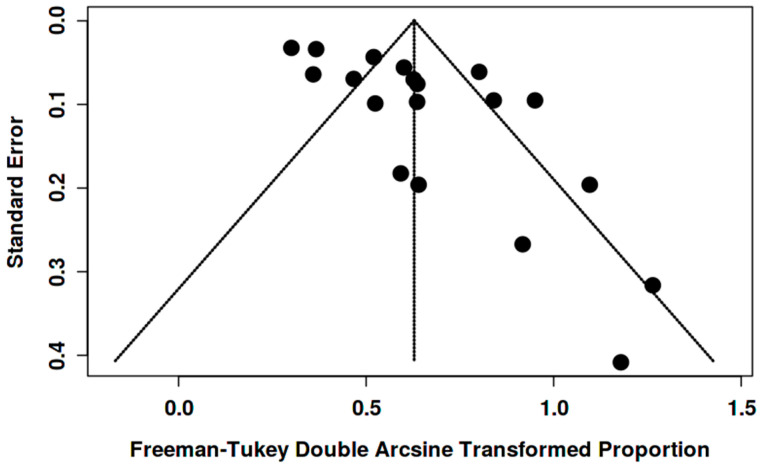
Funnel plot for overall risk of malignancy among BIUs (both genders).

Funnel plots showed asymmetry for the [^18^F]FDG “overall risk of malignancy” (Figure 7 and Figure 8). Egger’s test was significant in women (intercept 2.14, 95% CI 0.30–3.97; t = 2.286; *p* = 0.032) and in both genders (intercept 3.58, 95% CI 1.76–5.40; t = 3.848; *p* = 0.001), suggesting small-study effects/publication bias. Given the high heterogeneity and likely verification/selection bias in several retrospective studies, the funnel asymmetry should be interpreted with caution. It may reflect small-study effects/publication bias, but it could also come from center- and protocol-specific case-mix/work-up variation.

Taken together, BIU is uncommon on PET/CT but not rare, and a substantial fraction is malignant with tracer-dependent differences that likely mirror biological targets.

## 4. Discussion

### 4.1. Literature Data

To our knowledge, this is the most up-to-date systematic review with meta-analysis on breast incidental uptake (BIU) across multiple PET radiopharmaceuticals, extending earlier syntheses centered on [^18^F]FDG alone. We excluded case reports to minimize reporting/selection biases and because they typically lack an extractable denominator; consistent with methodological guidance for diagnostic/prognostic meta-analyses, we included only studies providing sufficient outcome data to compute summary estimates [8]. Our pooled results confirm that [^18^F]FDG-BIUs are uncommon but clinically meaningful: prevalence was 0.5% on all scans and 1.0% among women, while about 1/3 of BIUs in women proved malignant, consistent with prior meta-analytic results [5,6].

A key message from this multi-tracer review is the tracer-dependent difference. Although non-[^18^F]FDG evidence remains limited, somatostatin receptor (SSA) and [^18^F]fluorocholine series tended to show higher BIU prevalence and, among biopsied cases, higher malignancy proportions than [^18^F]FDG. For [^18^F]fluorocholine, the single eligible study in our dataset [38] reported BIU in 3.4% of women and malignancy among all biopsied BIUs (7/7); for SSAs, this systematic review highlights a higher malignancy risk for BIU than that for [^18^F]FDG. These results plausibly reflect biological targeting (receptor expression or phospholipid turnover) and background biodistribution. However, confidence intervals are wide given the small number of studies and events, so estimates should be interpreted with caution.

From a biological standpoint, lesion visibility on PET is influenced by histology and metabolic phenotype. [^18^F]FDG uptake is typically higher in ductal than in lobular carcinomas and correlates with tumor aggressiveness, which helps explain [^18^F]FDG-poor malignant BIUs in certain histotypes [39,40,41]. Our pooled histology mirrors prior reviews, with invasive ductal carcinoma predominating among malignant BIUs and fibroadenoma among benign outcomes [5]. Regarding SSTRs, they are overexpressed in many cancers, and increased choline uptake, driven by choline kinase activity, is also common in cancer cells. These differences in receptor expression help explain higher malignancy rates with SSA and [^18^F]fluorocholine tracers [4].

The literature on incidental second tumors also underscores that additional, clinically unsuspected malignancies are not rare in oncology populations, reinforcing the value of adequately investigating focal incidental uptake when encountered [42,43]. In practice, an incidental focal breast uptake on whole-body PET/CT should lead to targeted diagnostic breast imaging (mammography/ultrasound) and tissue sampling when indicated. Moreover, while some studies explore SUV cut-offs, SUV alone should not be used to differentiate between malignant and benign BIUs because SUV values for benign and malignant lesions often overlap and are affected by patient- and scanner-related factors, so SUV alone cannot accurately distinguish malignancy with the data currently available [5].

#### 4.1.1. Selection Bias in the Outlier Study

One notable outlier (Benveniste et al. [23]) reported BIU in nearly 1/4 [^18^F]FDG PET/CT examinations. This overestimation is due to the way the cohort was assembled: cases were identified by searching [^18^F]FDG PET/CT reports for the word “breast” instead of considering all scans. This keyword-based selection introduces bias, affecting prevalence estimates. For this reason, we included the paper in the qualitative discussion but excluded it from the pooled analysis [6,23].

#### 4.1.2. What to Do with an Incidental Focus

Because we found a meaningful risk of malignancy among BIUs, particularly with SSAs and fluorocholine, a structured management approach is warranted. In line with previous reviews, BIU should be further evaluated with mammography/breast ultrasound and with biopsy when indicated, while avoiding exclusive use of PET SUV-values alone [5].

### 4.2. Limitations and Suggestions for Future Research

This study has limitations. First, beyond [^18^F]FDG, the evidence base is small (only four SSA studies and one [^18^F]fluorocholine study), yielding wide CIs and susceptibility to small-study effects. Therefore, given the limited number of studies, these estimates should be interpreted cautiously and cannot yet inform clinical guidelines. Second, heterogeneity was substantial for several endpoints (e.g., BIU prevalence with [^18^F]FDG, overall malignancy risk), driven by differences in clinical indications (oncologic or non-oncologic indications, screening backgrounds), sex-specific denominators (often incomplete), verification bias (variable histology rates), and heterogeneity in data reporting (e.g., counts given per scan vs. per patient), and these aspects could explain the high I^2^. Third, verification bias: histology was available in a part of BIUs and loss to follow-up was common in retrospective designs, as also emphasized in prior BIU syntheses [5,6]. Fourth, selection biases (e.g., report-keyword filters) can markedly distort prevalence and malignancy proportions if included uncritically [23]. Fifth, evidence of funnel-plot asymmetry for [^18^F]FDG “overall risk of malignancy” suggests small-study effects or publication bias; this result should be interpreted cautiously. It may reflect small-study effects/publication bias, but it could also come from center- and protocol-specific case-mix/work-up variation.

Future work should ideally prospectively report PET/CT cohorts by tracer, with prospective registries and multicenter collaborations; use sex-specific denominators; adopt standardized BIU verification pathways; report histology for all worked-up BIUs (with reasons for non-verification); and integrate correlative pathology to clarify tracer-specific biology (e.g., SSTR expression, choline metabolism) and to assess subsequent management impact.

## 5. Conclusions

In clinical practice, [^18^F]FDG-BIUs are rare but meaningful, with malignancy found in about 1/3 of BIUs in women. Non-[^18^F]FDG tracers (SSAs and [^18^F]fluorocholine) show higher BIU prevalence and, among biopsied cases, higher malignancy proportions, but evidence is limited and confidence intervals are wide. BIU should lead to correlation with dedicated breast imaging and consideration of tissue sampling, while SUV-based discrimination is unreliable with the data currently available. Larger, standardized prospective studies, especially for SSAs and [^18^F]fluorocholine, are needed to refine prevalence and malignancy risk estimates and to define tracer-specific management pathways.

## Figures and Tables

**Table 1:** Prevalence and malignancy rate of breast incidental uptake detected by PET/CT with different tracers.

PET Tracer	First Author [Ref.]	Year	Country/ Study Type	No. of Scans (Both Genders)	Patients with BIU (Both Genders)	Prevalence of BIU on Scans (Both Genders)	No. of Scans (Female)	Patients with BIU (Female)	Prevalence of BIU on Scans (Female)	BIU-H	MBIU	Percentage of MBIU Among BIU-H	Overall Risk of Malignancy of BIU (Female)
**[^18^F]FDG**	Agress et al. [10]	2004	USA/R	1750	2	0.1%	NR	2	NC	2	2	100%	100%
	Ishimori et al. [11]	2005	USA/R	1912	7	0.4%	988	7	0.7%	2	2	100%	28.6%
	Korn et al. [12]	2006	USA/R	1098	6	0.5%	533	6	1.1%	6	5	83.3%	83.3%
	Wang et al. [13]	2007	Australia/R	1727	3	0.2%	747	3	0.4%	3	2	66.7%	66.7%
	Beatty et al. [14]	2009	USA/P	NR	NR	NC	1500	9	0.6%	8	5	62.5%	55.6%
	Litmanovich et al. [15]	2009	USA/R	NR	NR	NC	4038	33	0.8%	21	12	57.1%	36.4%
	Chung et al. [16]	2010	USA/R	45,000	60	0.1%	NR	60	NC	12	7	58.3%	11.7%
	Kang et al. [17]	2011	Korea/R	NR	NR	NC	13,897	50	0.4%	35	18	51.4%	36%
	Chae et al. [18]	2012	Korea/R	32,988	131	0.4%	NR	130	NC	60	32	53.3%	24.6%
	Chopra et al. [19]	2012	UK/R	818	6	0.7%	NR	6	NC	3	2	66.7%	33.3%
	Kim et al. [20]	2012	Korea/R	5214	27	0.5%	NR	26	NC	23	15	65.2%	57.7%
	Dunne et al. [21]	2013	Ireland/R	6050	50	0.8%	2719	50	1.8%	23	17	73.9%	34%
	Lim et al. [22]	2013	Korea/R	NR	NR	NC	7594	43	0.6%	17	9	52.9%	20.9%
	Benveniste et al. [23]	2015	USA/R	1951	438	22.5%	1866	438	23.5%	55	37	67.3%	8.4%
	Bertagna et al. [24]	2015	Italy/R	42,927	79	0.2%	NR	75	NC	35	25	71.4%	33.3%
	Minamimoto et al. [25]	2015	Japan/R	NR	NR	NC	62,054	473	0.8%	NR	161	NC	34%
	Shin et al. [26]	2015	Korea/R	55,762	214	0.4%	21,224	214	1%	60	27	45%	12.6%
	Falomo et al. [27]	2018	USA/R	8687	25	0.3%	NR	25	NC	11	6	54.5%	24%
	Moletta et al. [28]	2018	Italy/R	399	1	0.3%	NR	1	NC	1	1	100%	100%
	Bakhshayeshkaram et al. [29]	2019	Iran/R	5029	51	1%	NR	48	NC	22	10	45.5%	20.8%
	Andersen et al. [30]	2021	Denmark/R	19,551	66	0.3%	NR	62	NC	40	34	85%	54.8%
	Panareo et al. [31]	2021	Italy/R	3675	43	1.2%	NR	36	NC	22	15	68.2%	41.7%
	Wakfie-Corieh et al. [32]	2021	Spain/R	10,615	26	0.2%	NR	23	NC	23	9	39.1%	39.1%
	Menon et al. [33]	2023	Australia/R	5728	27	0.5%	NR	26	NC	26	18	69.2%	69.2%
	Kayadibi et al. [34]	2025	Turkey/R	12,633	234	1.9%	4263	234	5.5%	63	20	31.7%	8.5%
Pooled values (95%CI)						**0.5%** (0.3–0.6)			**1%** (0.6–1.6)			**60.9%** (52.5–69)	**33.5%** (25.2–42.3)
**Radiolabeled** **somatostatin analogues**	Elgeti et al. [35]	2008	Germany/R	33	4	12.1%	33	4	12.1%	4	4	100%	66.7%
	Kuyumcu et al. [36]	2013	Turkey/R	120	1	0.8%	63	1	1.6%	0	0	0%	0%
	Cavicchioli et al. [37]	2021	Brazil/P	41	1	2.4%	16	1	6.2%	1	1	100%	100%
Pooled values (95%CI)						**3.4%** (0.0–11.5)			**5.2%** (0.1–14.7)			**100%** (66.8–100)	**86.4%** (72–100)
**[^18^F]F-choline**	Broos et al. [38]	2022	Netherlands/R	388	10	2.6%	290	10	3.4%	7	7	100%	70%

Legend: BIU = breast incidental PET/CT tracer uptake, BIU-H = number of breast incidentalomas that underwent histology, MBIU = number of malignancies between breast incidentalomas, NC = not calculable, NR = not reported, R = retrospective, P = prospective.

## Data Availability

The original contributions presented in this study are included in the article. Further inquiries can be directed to the corresponding author.
