# Correction: Iacovitti et al. The Prevalence and Malignancy Risk of Breast Incidental Uptake Detected by PET/CT with Different Radiopharmaceuticals: An Updated Systematic Review and Meta-Analysis. *Pharmaceuticals* 2025, *18*, 1831

**DOI:** 10.3390/ph19010112

**Published:** 2026-01-08

**Authors:** Cesare Michele Iacovitti, Andreea Marin, Slavko Tasevski, Chiara Martinello, Marco Cuzzocrea, Gaetano Paone, Alessio Rizzo, Domenico Albano, Giorgio Treglia

**Affiliations:** 1 Division of Nuclear Medicine, Imaging Institute of Southern Switzerland, Ente Ospedaliero Cantonale, 6500 Bellinzona, Switzerland; cesaremichele.iacovitti@eoc.ch (C.M.I.); chiara.martinello@eoc.ch (C.M.); marco.cuzzocrea@eoc.ch (M.C.); gaetano.paone@eoc.ch (G.P.); 2 Clinic of Nuclear Medicine, Central University Emergency Military Hospital “Dr. Carol Davila”, 10825 Bucharest, Romania; andreea.marin0125@rez.umfcd.ro; 3 University Institute for Positron Emission Tomography, 1000 Skopje, North Macedonia; slavko.tasevski@uipet.mk; 4 Faculty of Biomedical Sciences, Università della Svizzera Italiana, 6900 Lugano, Switzerland; 5 Department of Nuclear Medicine, Candiolo Cancer Institute, FPO-IRCCS, 10060 Turin, Italy; alessio.rizzo@ircc.it; 6 Nuclear Medicine Department, ASST Spedali Civili, University of Brescia, 25123 Brescia, Italy; domenico.albano@unibs.it; 7 Faculty of Biology and Medicine, University of Lausanne, 1015 Lausanne, Switzerland

## Text Correction

There was an error in the original publication [1]. There is no mention of Figure S1 or Table S1 in the Supplementary Materials.

Corrections have been made to Section 2.1. Review Protocol, Working Group, and Review Question, Paragraph 1, and Section 3.1. Literature Search, Paragraph 1, as below:

“This review followed a predefined protocol [8] and is reported in accordance with PRISMA 2020 (PRISMA checklist reported in Table S1) [9].” 

“The selection process is summarized in Figures 1 and S1.”

## Missing Supplementary Materials

In the original publication [1], Supplementary Materials was not included. The Supplementary Materials part should read:

**Supplementary Materials:** The following supporting information can be downloaded at: https://www.mdpi.com/article/10.3390/ph18121831/s1, Figure S1: Flow-chart about the selection of studies according to PRISMA 2020. Table S1: PRISMA 2020 checklist.

The authors state that the scientific conclusions are unaffected. This correction was approved by the Academic Editor. The original publication has also been updated.
